# Reduced rate of intensive care unit acquired gram-negative bacilli after removal of sinks and introduction of ‘water-free’ patient care

**DOI:** 10.1186/s13756-017-0213-0

**Published:** 2017-06-10

**Authors:** Joost Hopman, Alma Tostmann, Heiman Wertheim, Maria Bos, Eva Kolwijck, Reinier Akkermans, Patrick Sturm, Andreas Voss, Peter Pickkers, Hans vd Hoeven

**Affiliations:** 1 Department of Medical Microbiology, Radboud university medical center, Geert Grooteplein 10, Postbus 9101, 6500 HB Nijmegen, The Netherlands; 2 Department of Medical Microbiology and Infectious Diseases, Canisius-Wilhelmina Hospital, Nijmegen, The Netherlands; 3 Department of Primary and Community Care, Radboud university medical center, Nijmegen, The Netherlands; 4 Department of Medical Microbiology, Laurentius hospital, Roermond, The Netherlands; 5 Department of Intensive Care, Radboud university medical center, Nijmegen, The Netherlands

**Keywords:** Intensive care unit, Sinks, Gram-negative bacilli, Multidrug resistance, ‘Water-free’ patient care, Length of stay, Colonization

## Abstract

**Background:**

Sinks in patient rooms are associated with hospital-acquired infections. The aim of this study was to evaluate the effect of removal of sinks from the Intensive Care Unit (ICU) patient rooms and the introduction of ‘water-free’ patient care on gram-negative bacilli colonization rates.

**Methods:**

We conducted a 2-year pre/post quasi-experimental study that compared monthly gram-negative bacilli colonization rates pre- and post-intervention using segmented regression analysis of interrupted time series data. Five ICUs of a tertiary care medical center were included. Participants were all patients of 18 years and older admitted to our ICUs for at least 48 h who also received selective digestive tract decontamination during the twelve month pre-intervention or the twelve month post-intervention period. The effect of sink removal and the introduction of ‘water-free’ patient care on colonization rates with gram-negative bacilli was evaluated. The main outcome of this study was the monthly colonization rate with gram-negative bacilli (GNB). Yeast colonization rates were used as a ‘negative control’. In addition, colonization rates were calculated for first positive culture results from cultures taken ≥3, ≥5, ≥7, ≥10 and ≥14 days after ICU-admission, rate ratios (RR) were calculated and differences tested with chi-squared tests.

**Results:**

In the pre-intervention period, 1496 patients (9153 admission days) and in the post-intervention period 1444 patients (9044 admission days) were included. Segmented regression analysis showed that the intervention was followed by a statistically significant immediate reduction in GNB colonization in absence of a pre or post intervention trend in GNB colonization. The overall GNB colonization rate dropped from 26.3 to 21.6 GNB/1000 ICU admission days (colonization rate ratio 0.82; 95%CI 0.67–0.99; *P* = 0.02). The reduction in GNB colonization rate became more pronounced in patients with a longer ICU-Length of Stay (LOS): from a 1.22-fold reduction (≥2 days), to a 1.6-fold (≥5 days; *P* = 0.002), 2.5-fold (for ≥10 days; *P* < 0.001) to a 3.6-fold (≥14 days; *P* < 0.001) reduction.

**Conclusions:**

Removal of sinks from patient rooms and introduction of a method of ‘water-free’ patient care is associated with a significant reduction of patient colonization with GNB, especially in patients with a longer ICU length of stay.

**Electronic supplementary material:**

The online version of this article (doi:10.1186/s13756-017-0213-0) contains supplementary material, which is available to authorized users.

## Background

Hospital acquired infections in the Intensive Care Unit (ICU) result in patient morbidity and mortality [1]. Environmental contamination in hospitals wards and ICUs is a recognized problem for infection prevention and control [2–7], as the environment may facilitate transmission of several important health care-associated pathogens, including gram-negative bacilli (GNB) [8]. As part of the traditional hospital hand hygiene strategy and patient care, sinks are present in virtually all hospital wards and patient rooms. While sinks in the proximity of patients are advocated as a best practice of ICU design [9], involvement of these sinks in hospital-associated infections have been reported as early as the 1970s [10–14]. Recent publications have highlighted the role of sinks as a source of outbreaks and transmission of multidrug-resistant gram-negative bacilli (MDR-GNB) in intensive care units, including paediatric and neonatal ICUs [15–28]. Interventions to reduce transmission of MDR-GNB from sinks in outbreak settings have been explored [29–31], while the effect of sinks on overall infection and colonization rates has not been studied.

As multi-drug resistance (MDR) in GNB is an increasing problem in the management of hospitalized patients [32–34], we investigated the effect of the removal of sinks from the ICU patient rooms combined with ‘water-free’ patient care on ICU-acquired GNB colonization rates in patients admitted to the ICU.

## Methods

### Background and study design

In early 2014 an outbreak with extended-spectrum β-lactamase (ESBL)-producing *Enterobacter cloacae* was identified in our ICU that could be related to contaminated sinks. When the decision to remove the sinks and to implement the ‘water-free’ patient care method was taken, it was prospectively decided to evaluate its effect after 12 months. We conducted a pre/post quasi-experimental study to evaluate the effect of sink removal and introduction of ‘water-free’ patient care on colonization with GNB in patients admitted to the ICU for at least 48 h during a 12-month pre-intervention (May 2013–April 2014), the months of intervention (May 2014–August 2014) and a 12-month post-intervention period (September 2014–August 2015).

### Study setting

This study was conducted in a large tertiary care medical center in the Netherlands with 953 beds. The ICU consists of five subunits, with a total 34 operational single patient rooms. Patients admitted to the ICU that need mechanical ventilation and are anticipated to stay >24 h receive selective digestive tract decontamination (SDD), which consists of 4 days of intravenous cefotaxime and topical application of tobramycin, colistin, and amphotericin B in the oropharynx and stomach [35]. No alterations were made to the SDD protocol during the study period. An essential part of SDD strategy is twice a week routine screening for colonization with gram-negative bacilli and yeasts from rectal, sputum and throat swabs.

### The intervention

Between May and August 2014, all sinks were removed from all ICU patient rooms and a ‘water-free’ method of patient care was introduced, meaning that all patient care related activities that take place in the patient room and that would normally involve the use of tap water were adapted to a ‘water-free’ alternative, see Table 1.

**Table 1:** ‘Water-free’ patient care activities

Patient care-related action	New method with ‘water-free’ working
Gloves and gowns	Universal gloving and gowning (pre- and post-intervention period)
Hand washing after visual contamination	‘Quick & Clean’, (Alpheios B.V., Heerlen, The Netherlands) wipes to remove extensive contamination from hands. Followed by disinfection with alcohol-based hand rub
Medication preparation	Dissolving of medication in bottled water (SPA reine, Spa, Belgium)
Drinks	Bottled water (SPA reine, Spa, Belgium)
Canula care	Disposable materials
Hair washing	Rinse-free shampoo cap (Comfort Personal cleansing products, USA)
Washing	Moistened disposable wash gloves, (D-care,Houten, The Netherlands)
Dental care	Bottled (SPA reine, Spa, Belgium)
Shaving	Electric shaving, or with warm bottled water (SPA reine, Spa, Belgium)

### Patient selection and medical ethical aspects

All patients of 18 years and older who were admitted to the ICU for at least 48 h were included in this study. The study was reviewed and approved (File number CMO: 2015–1764) by the ethics committee of the Radboud university medical centre and was carried out in accordance with the applicable rules concerning biomedical research using patient information. Patient data were collected and analyzed anonymously.

### Data collection

Data were collected in a standardized manner according to standard definitions and were subject to data quality checks [36]. Demographic information including sex and age, referring specialty and location before ICU admission, type of admission, comorbidity, Acute Physiology and Chronic Health Evaluation (APACHE) score, days on mechanical ventilation, and ICU length of stay, were collected. We collected culture results (from routine SDD screenings) from the medical microbiology laboratory database. Culture results from cultures taken <48 h of admission, including all repeat findings, were excluded from further analyses. When a patient was readmitted to the ICU during the study period, culture results identical to the first ICU admission were excluded.

### Outcomes and definitions

The primary outcome of this study was the GNB colonization rate, calculated as the number of primary positive microbiological results per 1000 ICU admission days, during the pre- and post-intervention periods. The colonization rates of patients with yeasts were used as a ‘negative control’, as yeasts do not thrive in sinks and the ICU sinks at all times had been free of yeast colonization.

### Statistical analysis

‘To compare the patient characteristics between pre-intervention and post-intervention period, we described continuous data as mean ± standard deviation and groups were compared using a Student-t-test, or as median (25th and 75th percentile) and compared using a Mann-Whitney U test, depending on the distribution. Dichotomous or categorical data were described as number with percentage and subgroups were compared using a Chi-squared test. The pre- and post-intervention GNB and yeast colonization rates were calculated per 1000 admission days. The colonization rate ratios were (with 95% confidence interval (CI)) calculated to quantify the effect of the intervention on these rates. For calculating the subsequent colonization rates related to ICU- length of stay (LOS), only admissions of ≥3, ≥5, ≥7, ≥10 or ≥14 days were used for the denominator (number of admission days).

Segmented regression analysis of interrupted time series data was conducted to estimate the effect of the intervention on the monthly GNB and yeast colonization rates, both immediately and over time and to identify whether there was a baseline or a post-intervention monthly trend in colonization rate [37]. An autoregressive integrated moving average (ARIMA) model was used. The model was adjusted for negative first order autocorrelation by including an autocorrelation parameter in the segmented regression model [37]. To determine if colonization was likely to be ICU-acquired and to relate it to exposure duration, time-dependent (ICU-LOS) effects were investigated. In this ARIMA model, β_0_ estimates the baseline level of the monthly colonization rate at time zero; β_1_ estimates the pre-intervention or baseline linear trend of the monthly colonization rate; β_2_ estimates the level change in the monthly colonization rate immediately after the intervention (i.e. step change or change in level: immediate effect of the intervention); and β_3_ estimates the post-intervention change in linear trend of the monthly colonization rate. Predicted rates are calculated based on model parameters. The rates during the intervention months (May 2014 – August 2014) were excluded from this analysis.

First, the full regression model was specified for the GNB and the yeast colonization, meaning that the following estimates were given: β_0_, β_1_, β_2_, and β_3_. After stepwise elimination of non-significant terms, the most parsimonious model contained only the intercept (β_0_) and the significant level change (β_2_) in the monthly colonization rate. This segmented regression analysis was performed on all GNB identified ≥2 days after ICU admission, and subsequently repeated for GNB first identified ≥3, ≥5, ≥7, ≥10 or ≥14 days after ICU admission, respectively.

If the segmented regression analysis would show that there was no monthly trend in GNB colonization either before or after the intervention, overall GNB colonization rates were calculated and compared between pre- and post-intervention and were defined as the number of GNB (or MDR-GNB) per 1000 ICU admission days. The rates during the intervention months (May 2014 – August 2014) were excluded from this analysis. Colonization rate ratios (and 95% confidence intervals) were calculated to quantify the effect of the intervention on the outcome and rates were compared using a Chi-squared test. This analysis was repeated for GNB identified ≥3, ≥5, ≥7, ≥10 or ≥14 days after ICU admission, respectively.

Statistical analysis was performed using IBM SPSS Statistics version 22 and STATA/SE version 11.0. A two-sided *p*-value <0.05 was considered to indicate statistical significance.

## Results

An increased number of *Enterobacter cloacae* ESBL positive isolates was detected and communicated to the ICU in May 2014. In total 11 isolates pre and one isolate post-intervention were identified. By molecular typing we were able to show that 5 isolates were related pre-intervention. Sinks in the ICU were tested positive for *Enterobacter cloacae* ESBL prior to removal. The outbreak developed despite routine use of extensive infection prevention measures including the use of protective clothing and gloves with all patient contacts. It was decided to remove the sinks from all ICU patient rooms in order to eradicate the source of MDR-GNB in the direct patient environment.

**Fig. 1:**
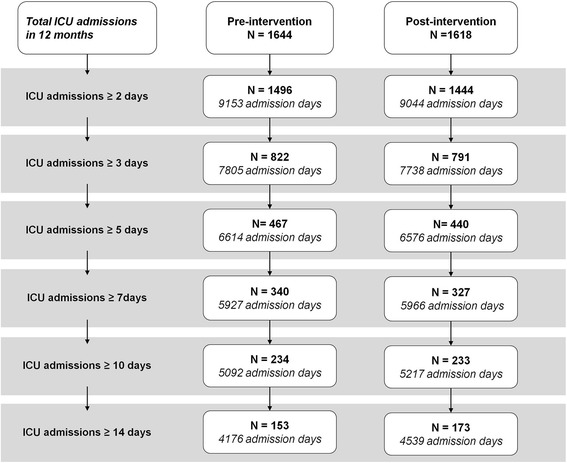
Flow chart ICU admissions. Legend: Flowchart of the number of patients with an ICU-length of stay of ≥2 days, ≥3 days, ≥5 days, ≥7 days, ≥10 days and ≥14 days, and the subsequent number of admission days

1644 patients were admitted to the ICU in the 12 months prior to the removal of sinks from the ICU patient rooms, of which 1496 patients had a ICU-LOS ≥2 days (total 9153 admission days). In the 12 months after the removal of sinks, 1618 patients were admitted to the ICU, of which 1444 were in the ICU for ≥2 days (total 9044 admission days). 145 (9.7%) in the pre-intervention period and 137 (9.5%) post-intervention were re-admissions (*P* = 0.85). See Fig. 1.

The baseline demographic characteristics of the patients at ICU admission are described in Table 2. Apart from a statistically significant difference between pre- and post-intervention patients for chronic respiratory insufficiency as a comorbidity, no other relevant differences in demographics were observed. The median ICU-length of stay was 3 days (IQR 2–6 days) pre-intervention, and 3 days (IQR 2–6 days) post-intervention (*p* = 0.90). In the pre- and post-intervention periods, 31.2% and 30.5% (*P* = 0.66) had an ICU-LOS ≥5 days, and 15.6% and 16.1% (*P* = 0.71) had an ICU-LOS ≥ 10 days, respectively. Over a third of the ICU admissions (38.3% pre-intervention; 34.9% post-intervention; *P* = 0.06) had a type of registered comorbidity at admission. A statistically significant difference between pre- and post-intervention patients (7.8% vs 4.9%, respectively; *P* = 0.002) was observed for chronic respiratory insufficiency.

**Table 2:** Characteristics of ICU admissions of ≥2 days before and after sink removal

	Pre intervention		Post intervention		
	*n*	%	*n*	%	*P*-value
ICU admissions with LOS of ≥48 h	*N* = 1496		*N* = 1444		
First or re-admission					
Primary admissions	1351	90.3%	1307	90.5%	0.85
Re-admissions	145	9.7%	137	9.5%	
Age, median (IQR)	62	[50–70]	63	[52–71]	0.07
Male sex, *n* (%)	890	59.5%	856	59.4%	0.94
BMI, mean (SD)	26.1	5.3	26.3	5.2	0.31
ICU mortality, *n* (%)	174	11.6%	146	10.1%	0.19
Hospital mortality, *n* (%)	225	15.0%	207	14.3%	0.59
ICU Lenght of stay (LOS), median days (IQR)	3	[2–6]	3	[2–6]	0.90
ICU LOS, *n* (%)					
2 days	674	45.1%	653	45.2%	0.38
3–4 days	355	23.7%	351	24.3%	
5–6 days	127	8.5%	113	7.8%	
7–9 days	106	7.1%	94	6.5%	
10–13 days	81	5.4%	60	4.2%	
≥ 14 days	153	10.2%	173	12.0%	
Apache score, mean (SD)	18.7	7.2	18.2	7.2	0.27
Days on respirator, median (IQR)	2	[0–4]	1	[0–4]	0.38
Comorbidity at ICU admission, *n* ‘yes’ (%)					
Any comorbidity	573	38.3%	504	34.9%	0.06
Cardiovascular insufficiency	93	6.2%	70	4.8%	0.11
Respiratory insufficiency	116	7.8%	71	4.9%	0.002
Diabetes	180	12.0%	168	11.6%	0.74
Chronic renal insufficiency	97	6.5%	83	5.7%	0.41
Neoplasm	130	8.7%	112	7.8%	0.36
Immune-insufficiency	166	11.1%	166	11.5%	0.93
Medical specialty, *n* (%)					
Surgery	330	22.1%	361	25.0%	0.04
Neurosurgery	239	16.0%	200	13.9%	
Thoracic surgery	234	15.6%	245	17.0%	
Pulmonary disease	125	8.4%	139	9.6%	
Internal medicine	64	4.3%	72	5.0%	
Other	504	33.7%	427	29.6%	
Admission type, *n* (%)					
Medical	732	48.9%	712	49.3%	0.06
Elective	528	35.3%	464	32.1%	
Emergency	236	15.8%	268	18.6%	
Admission source, *n* (%)					
Emergency	372	24.9%	344	23.8%	0.27
Clinical department	292	19.5%	302	20.9%	
Other IC unit	93	6.2%	69	4.8%	
Other	739	49.4%	729	50.5%	

### Interrupted time series analysis

**Fig. 2:**
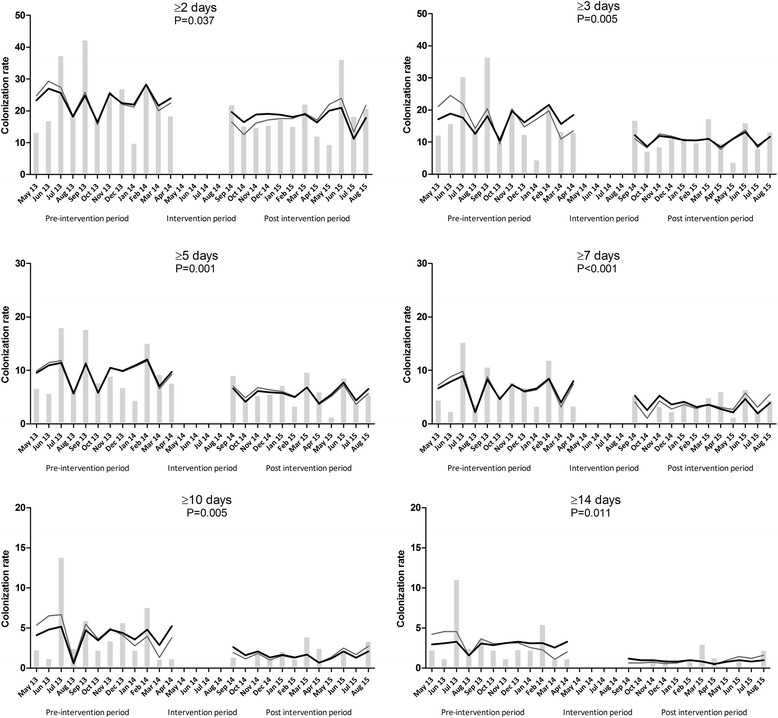
Monthly gram-negative bacilli (GNB) colonization rates. Legend: Monthly GNB colonization rates (bars), the predicated rate based on the full model (*grey line*) and the predicted rate based on the parsimonious model (*black line*). β2 level change *p*-values are shown in 2A to 2F, where β2 stands for the level change in the monthly colonization rate immediately after the intervention. Section A to F refer to GNB identified in ICU patients with a length of stay of ≥2, ≥3, ≥5, ≥7, ≥10 or ≥14 days after ICU admission

The results of the segmented regression analysis are shown in Additional file 1: Table S1. There was a statistically significant immediate effect of the removal of sinks on the monthly colonization rate of GNB, but not on the colonization rate of yeasts, with statistically significant β2 level changes for all GNB colonization outcomes for the different ICU LOS (*P* = 0.037 for ICU LOS ≥48 h, *P* = 0.005 for ICU LOS ≥3 days; *P* = 0.001 for ICU LOS ≥5 days; *P* < 0.001 for ICU LOS ≥7 days; *P* = 0.005 for ICU LOS ≥10 days; *P* = 0.011 for ICU LOS ≥14 days) . There was no pre-intervention drift in monthly GNB rates and this was also the case in the ICU-LOS-dependent analyses. Graphs with the observed and predicted colonization rates are shown in Fig. 2. The data for the interrupted time series analysis for yeast colonization are shown in Additional file 2: Figure S4.

In the most parsimonious model, the pre-intervention trend (β_1_) and post-intervention trend-change (β_3_) were omitted, resulting in a statistically significant immediate effect of the intervention on the GNB colonization rates.

### Overall GNB colonization rates

**Fig. 3:**
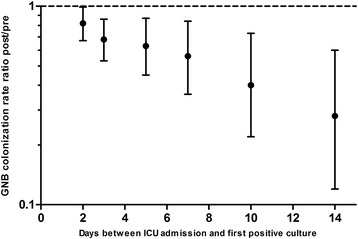
Colonization rate ratios related to ICU-LOS. Legend: Colonization rate ratios (with 95%CI) were calculated to investigate the effect of ICU-LOS on the effect of the intervention. GNB identified in ICU patients with a length of stay of ≥2, ≥3, ≥5, ≥7, ≥10 or ≥14 days after ICU admission were analyzed

The overall GNB colonization rates were 26.3 and 21.6 GNB/1000 ICU admission days (rate ratio 0.82; 95%CI 0.67–0.99; *P* = 0.02) for pre- and post-intervention groups, respectively. The difference between the groups became more pronounced over time: GNB colonization rates that were first identified in cultures taken ≥3 days (22.5 vs. 15.2; RR 0.68; 95%CI 0.53–0.86; *P* < 0.001), cultures taken ≥5 days (15.0 vs. 9.4; RR 0.63; 95%CI 0.45–0.87; *P* = 0.002), cultures taken ≥7 days (11.5 vs. 6.4; RR 0.56; 95%CI 0.36–0.84; *P* = 0.002), cultures taken ≥10 days (8.1 vs. 3.3; RR 0.40; 95%CI 0.22–0.73; *P* < 0.001) and cultures taken ≥14 days after ICU admission (7.2 vs. 2.0; RR 0.28; 95%CI 0.12–0.60; *P* < 0.001). As also illustrated by Fig. 3, the effect of the intervention in the GNB colonization rate increases with increasing LOS on patients at the ICU.

The (MDR-)GNB that were found on all time points are summarized in Additional file 3: Table S2.

## Discussion

We have shown that the removal of sinks in patient rooms and implementation of water-free patient care is associated with a significant reduction of patient colonization with GNB and this effect was most pronounced in patients with a longer ICU length of stay.

The effect of the intervention on GNB colonization rates became even more apparent when pathogens that were first identified after longer durations of ICU stay were compared between the pre-intervention and post-intervention period. Apart from the fact that with increased ICU-LOS the likelihood increases that these pathogens were acquired at the ICU, it appears plausible that a longer stay in an ICU increases the exposure to potential pathogens in the direct patient surroundings including those originated from the sinks.

The lower number GNB in the post-intervention period cannot be explained by an overall decrease in observed pathogens, as there was no effect of the intervention on yeast colonization rates. Yeast do not thrive in sinks or siphons and therefore we used them as a negative control. Furthermore, the overall number of cultures processed in the pre- and post-intervention study period were similar meaning that there was no reduction in the total number of screening cultures taken that could explain our findings.

In this study, we focused on colonization rates, and not infections. Even though infections caused by GNB would have been a more relevant clinical outcome than colonization, demonstrating the effect of an intervention on clinical infection rate would require a sample size that is not feasible. Previous work on the effects of SDD on infections with gram-negative bacilli showed that the cumulative incidence of ICU-acquired bacteremia in the SDD study group was 0.9%. To demonstrate a 30% reduction related to this intervention, approximately 26,000 patients would need to be included. Nevertheless, as colonization precedes infection, it is plausible that the intervention will have an impact on bloodstream infections with GNB.

### Limitations of the study

Several limitations of this study need to be addressed. First and most importantly, this is an open label, non-randomized single-centre study. Naturally, the implementation procedures importantly limited the feasibility of using other study designs. Despite of the design limitations, in the absence of alternative explanations, we believe that it is conceivable that the removal of sinks and implementation of water-free patient care resulted in a significant reduction of GNB colonization. There was no pre-existing downward drift in colonization rate, no changes were made during the study period in the hand hygiene protocol, protocol of standard or transmission-based precautions and the protocols of cleaning and disinfection. No chlorhexidine gluconate bathing is performed in our ICU. The quality of cleaning and disinfection remained constant and antibiotic guidelines did not alter during the study. The only difference between the pre- and post-intervention periods were the differences in some of the baseline demographic characteristics, e.g., patients in the pre-intervention period more often suffered chronic respiratory insufficiency compared to post-intervention admissions. However, as the vast majority (87%) of GNB colonization was identified in patients without chronic respiratory insufficiency, it appears unlikely that this difference could account for the observed effects. Importantly, no relevant changes in procedures, staffing levels, technical infrastructure, or other major changes that could influence patient management took place during the conduct of the study. No alternative confounders could be identified that could have influenced the outcome of the study. Second, our intervention was performed in a relatively “low GNB endemic setting” due to the use of SDD [35]. It is difficult to predict how the findings of this study can be generalized to a broader setting including non-SDD hospitals. On ICU’s with a higher GNB colonization rate compared to our setting, it appears plausible that the effects could be more pronounced. Removing sinks from patient rooms could be a very effective intervention with a high impact for ICUs in low-resource settings, where nosocomial infections with GNB are very common [38]. Some may argue that the removal of the sinks could interfere with the prevention of nosocomial transmission of *Clostridium difficile,* as spores are resistant to alcohol-based handrub*.* In our hospital the incidence of *Clostridium difficile* infections is very low. Over the last 2.5 years 4 patients were diagnosed with *Clostridium difficile* in the ICU. Centers for Disease Control and Prevention advises to wear gloves when caring for patients with *C. difficile*-associated diarrhea. After gloves are removed, hands should be washed with a non-antimicrobial or an antimicrobial soap and water or disinfected with an alcohol-based handrub [39]. Our ICU setting with use of gloves in all patient contacts is in line with these recommendations. In our ICU, we have purchased a mobile hand washing sink that can be used as a back-up in case of a serious *Clostridium* infection outbreak.

In view of our results we should reconsider the necessity of sinks and other ‘wet’ areas in the patient rooms. Under time constraints, healthcare workers compliance with infection prevention and control measures is often reduced, specifically in the case of hand hygiene, infection prevention protocols and waste management protocols. Reconstructing the hospital infrastructure in a way that behavior of healthcare workers is more directed towards good clinical practice is a step in the direction of sustainable infection control.

## Conclusions

This study shows that removal of the sinks from all patient rooms and the introduction of ‘water-free’ patient care is associated with a statistically significant lower number of ICU patients that become colonized with GNB, including MDR-GNB, especially among patients with a longer length of stay at the ICU. To our knowledge, this is the first study that indicates that sinks in patient rooms not only play a role in outbreak situations, but also in sporadic transmission of GNB from sinks to patients.

## Additional files

Additional file 1: Segmented regression models predicting GNB (A) and yeast (B) colonization rates. (DOCX 28 kb)

Additional file 2: (JPEG 5541 kb)

Additional file 3: Colonization with Gram-negative bacilli. (DOCX 28 kb)
