# Supplementary material for: Reduced rate of intensive care unit acquired gram-negative bacilli after removal of sinks and introduction of ‘water-free’ patient care
Source: Antimicrob Resist Infect Control. 2017 Jun 10;6:59. doi: 10.1186/s13756-017-0213-0 (PMC5466749; doi:10.1186/s13756-017-0213-0)
Supplement: Supplementary file 1 — Segmented regression models predicting GNB (A) and yeast (B) colonization rates. (DOCX 28 kb) [file 13756_2017_213_MOESM1_ESM.docx]

**Additional file 1:Table S1. Segmented regression models predicting GNB (A) and yeast (B) colonization rates.**

**eTable 3A: GNB colonization**

| **Full interrupted time series model** |  |  |  |  |  | **Most parsimonious model** | | |  |
| --- | --- | --- | --- | --- | --- | --- | --- | --- | --- |
|  | **Estimate** | **SE** | **t** | **Sig.** |  | **Estimate** | **SE** | **t** | **Sig.** |
|  |  |  |  |  |  |  |  |  |  |
| **GNB ≥48h after ICU admission** |  |  |  |  |  |  |  |  |  |
| **Base level, β_0_** | 21.797 | 3.651 | 5.971 | 0.000 |  | 23.248 | 1.699 | 13.680 | 0.000 |
| Pre-intervention trend**, β_1_** | -0.238 | 0.503 | -0.474 | 0.641 |  |  |  |  |  |
| **Level change, β_2_** | -7.407 | 5.334 | -1.389 | 0.181 |  | -5.409 | 2.425 | -2.231 | 0.037 |
| Trend change post intervention**, β_3_** | 0.773 | 0.693 | 1.115 | 0.279 |  |  |  |  |  |
|  |  |  |  |  |  |  |  |  |  |
| **GNB ≥3 days after ICU admission** |  |  |  |  |  |  |  |  |  |
| **Base level, β_0_** | 12.493 | 2.917 | 4.283 | 0.000 |  | 17.103 | 1.455 | 11.753 | 0.000 |
| Pre-intervention trend**, β_1_** | -0.721 | 0.402 | -1.792 | 0.089 |  |  |  |  |  |
| **Level change, β_2_** | -1.645 | 4.268 | -0.385 | 0.704 |  | -6.453 | 2.076 | -3.109 | 0.005 |
| Trend change post intervention**, β_3_** | 0.697 | 0.554 | 1.257 | 0.224 |  |  |  |  |  |
|  |  |  |  |  |  |  |  |  |  |
| **GNB ≥5 days after ICU admission** |  |  |  |  |  |  |  |  |  |
| **Base level, β_0_** | 9.107 | 1.557 | 5.849 | 0.000 |  | 9,549 | 0,681 | 14,019 | 0.000 |
| Pre-intervention trend**, β_1_** | -0.067 | 0.215 | -0.312 | 0.759 |  |  |  |  |  |
| **Level change, β_2_** | -2.738 | 2.279 | -1.202 | 0.244 |  | -3,893 | 0,972 | -4,003 | 0.001 |
| Trend change post intervention**, β_3_** | -0.042 | 0.296 | -0.143 | 0.888 |  |  |  |  |  |
|  |  |  |  |  |  |  |  |  |  |
| **GNB ≥7 days after ICU admission** |  |  |  |  |  |  |  |  |  |
| **Base level, β_0_** | 6.062 | 1.133 | 5.350 | 0.000 |  | 6.652 | 0.529 | 12.564 | 0.000 |
| Pre-intervention trend**, β_1_** | -0.096 | 0.157 | -0.614 | 0.547 |  |  |  |  |  |
| **Level change, β_2_** | -3.867 | 1.662 | -2.326 | 0.031 |  | -3.205 | 0.757 | -4.236 | 0.000 |
| Trend change post intervention**, β_3_** | 0.292 | 0.215 | 1.355 | 0.191 |  |  |  |  |  |
|  |  |  |  |  |  |  |  |  |  |
| **GNB ≥10 days after ICU admission** |  |  |  |  |  |  |  |  |  |
| **Base level, β_0_** | 2.653 | 1.196 | 2.218 | 0.039 |  | 4.091 | 0.571 | 7.168 | 0.000 |
| Pre-intervention trend**, β_1_** | -0.225 | 0.165 | -1.367 | 0.187 |  |  |  |  |  |
| **Level change, β_2_** | -1.578 | 1.748 | -0.903 | 0.378 |  | -2.583 | 0.816 | -3.167 | 0.005 |
| trend change post intervention**, β_3_** | 0.294 | 0.227 | 1.297 | 0.210 |  |  |  |  |  |
|  |  |  |  |  |  |  |  |  |  |
| **GNB ≥14 days after ICU admission** |  |  |  |  |  |  |  |  |  |
| **Base level, β_0_** | 1.535 | 1.051 | 1.460 | 0.161 |  | 2.958 | 0.528 | 5.607 | 0.000 |
| Pre-intervention trend**, β_1_** | -0.222 | 0.144 | -1.540 | 0.140 |  |  |  |  |  |
| **Level change, β_2_** | -1.174 | 1.526 | -0.769 | 0.451 |  | -2.112 | 0.752 | -2.807 | 0.011 |
| Trend change post intervention**, β_3_** | 0.298 | 0.200 | 1.490 | 0.153 |  |  |  |  |  |

**eTable 3B. Yeast colonization**

|  |  |  |  |  |  |  | | |  |
| --- | --- | --- | --- | --- | --- | --- | --- | --- | --- |
| **Full interrupted time series model** |  |  |  |  |  | **Most parsimonious model** | | |  |
|  |  |  |  |  |  |  |  |  |  |
|  | **Estimate** | **SE** | **t** | **Sig.** |  | **Estimate** | **SE** | **t** | **Sig.** |
|  |  |  |  |  |  |  |  |  |  |
| **Yeasts ≥48h after ICU admission** |  |  |  |  |  |  |  |  |  |
| **Base level, β_0_** | 23.210 | 3.376 | 6.874 | 0.000 |  | 20.962 | 1.564 | 13.404 | 0.000 |
| Pre-intervention trend**, β_1_** | 0.348 | 0.457 | 0.761 | 0.456 |  |  |  |  |  |
| **Level change, β_2_** | -5.524 | 4.719 | -1.171 | 0.256 |  | -1.435 | 2.203 | -0.652 | 0.522 |
| Trend change post intervention**, β_3_** | -0.062 | 0.653 | -0.095 | 0.925 |  |  |  |  |  |
|  |  |  |  |  |  |  |  |  |  |
| **Yeasts ≥3 days after ICU admission** |  |  |  |  |  |  |  |  |  |
| **Base level, β_0_** | 17.615 | 2.905 | 6.063 | 0.000 |  | 16.058 | 1.313 | 12.228 | 0.000 |
| Pre-intervention trend**, β_1_** | 0.241 | 0.393 | 0.612 | 0.548 |  |  |  |  |  |
| **Level change, β_2_** | -4.982 | 4.042 | -1.232 | 0.233 |  | -2.688 | 1.852 | -1.452 | 0.161 |
| Trend change post intervention**, β_3_** | -0.126 | 0.562 | -0.225 | 0.825 |  |  |  |  |  |
|  |  |  |  |  |  |  |  |  |  |
| **Yeasts ≥5 days after ICU admission** |  |  |  |  |  |  |  |  |  |
| **Base level, β_0_** | 12.235 | 2.486 | 4.922 | 0.000 |  | 7.722 | 1.460 | 5.289 | 0.000 |
| Pre-intervention trend**, β_1_** | 0.718 | 0.334 | 2.151 | 0.045 |  |  |  |  |  |
| **Level change, β_2_** | -6.705 | 3.352 | -2.000 | 0.060 |  | -2.323 | 2.063 | -1.126 | 0.273 |
| Trend change post intervention**, β_3_** | -0.692 | 0.484 | -1.430 | 0.169 |  |  |  |  |  |
|  |  |  |  |  |  |  |  |  |  |
| **Yeasts ≥7 days after ICU admission** |  |  |  |  |  |  |  |  |  |
| **Base level, β_0_** | 4.948 | 1.382 | 3.580 | 0.002 |  | 3.375 | 0.745 | 4.529 | 0.000 |
| Pre-intervention trend**, β_1_** | 0.239 | 0.187 | 1.280 | 0.216 |  |  |  |  |  |
| **Level change, β_2_** | -0.773 | 1.924 | -0.402 | 0.692 |  | -0.356 | 1.039 | -0.342 | 0.736 |
| Ttrend change post intervention**, β_3_** | -0.415 | 0.267 | -1.556 | 0.136 |  |  |  |  |  |
|  |  |  |  |  |  |  |  |  |  |
| **Yeasts ≥10 days after ICU admission** |  |  |  |  |  |  |  |  |  |
| **Base level, β_0_** | 3.772 | 1.022 | 3.692 | 0.002 |  | 2.111 | 0.577 | 3.660 | 0.001 |
| Pre-intervention trend**, β_1_** | 0.259 | 0.138 | 1.876 | 0.076 |  |  |  |  |  |
| **Level change, β_2_** | -2.539 | 1.418 | -1.791 | 0.089 |  | -0.784 | 0.801 | -0.979 | 0.339 |
| Trend change post intervention**, β_3_** | -0.238 | 0.197 | -1.211 | 0.241 |  |  |  |  |  |

**Legend:** Parameter estimates, standard errors and P-values from the full and most parsimonious segmented regression models predicting mean monthly gram-negative bacilli (A) and yeast (B) colonization rates.
