# Supplementary material for: Reduced rate of intensive care unit acquired gram-negative bacilli after removal of sinks and introduction of ‘water-free’ patient care
Source: Antimicrob Resist Infect Control. 2017 Jun 10;6:59. doi: 10.1186/s13756-017-0213-0 (PMC5466749; doi:10.1186/s13756-017-0213-0)
Supplement: Supplementary file 3 — Colonization with Gram-negative bacilli. (DOCX 28 kb) [file 13756_2017_213_MOESM3_ESM.docx]

**Addiitonal file 3: Table S2. Colonization with Gram-negative bacilli.**

| **All GNBs ≥ 2 days** | **Control** | **Intervention** | **All GNBs** ≥ 3 days | **Control** | **Intervention** |
| --- | --- | --- | --- | --- | --- |
| Achromobacter species | 0 | 2 | Achromobacter species | 0 | 2 |
| Acidovorax species | 2 | 0 | Acidovorax species | 2 | 0 |
| Acinetobacter baumannii | 1 | 3 | Acinetobacter baumannii | 0 | 1 |
| Acinetobacter baumannii BRMO | 0 | 1 | Acinetobacter baumannii BRMO | 0 | 1 |
| Acinetobacter calcoaceticus | 0 | 1 | Acinetobacter pittii | 0 | 1 |
| Acinetobacter pittii | 0 | 1 | Alcaligenes faecalis | 1 | 0 |
| Alcaligenes faecalis | 1 | 0 | Burkholderia cepacia complex | 1 | 0 |
| Burkholderia cepacia complex | 1 | 0 | Chryseobacterium gleum | 0 | 2 |
| Chryseobacterium gleum | 0 | 2 | Chryseobacterium indologenes | 3 | 0 |
| Chryseobacterium indologenes | 3 | 0 | Citrobacter freundii | 1 | 3 |
| Citrobacter freundii | 1 | 3 | Citrobacter freundii BRMO | 0 | 1 |
| Citrobacter freundii BRMO | 0 | 1 | Citrobacter species | 3 | 1 |
| Citrobacter species | 4 | 1 | Citrobacter species BRMO | 1 | 0 |
| Citrobacter species BRMO | 1 | 0 | Citrobacter species ESBL | 2 | 0 |
| Citrobacter species ESBL | 2 | 0 | Delftia | 1 | 0 |
| Comamonas species | 0 | 1 | Delftia acidovorans | 1 | 1 |
| Delftia | 1 | 0 | Elizabethkingia species | 2 | 0 |
| Delftia acidovorans | 1 | 1 | Enterobacter aerogenes | 1 | 4 |
| Elizabethkingia species | 2 | 0 | Enterobacter cloacae complex | 15 | 8 |
| Enterobacter aerogenes | 1 | 7 | Enterobacter cloacae complex BRMO | 1 | 0 |
| Enterobacter cloacae | 1 | 0 | Enterobacter cloacae complex ESBL | 10 | 1 |
| Enterobacter cloacae complex | 19 | 8 | Escherichia coli | 46 | 38 |
| Enterobacter cloacae complex BRMO | 1 | 0 | Escherichia coli AmpC | 1 | 0 |
| Enterobacter cloacae complex ESBL | 11 | 1 | Escherichia coli BRMO | 3 | 3 |
| Escherichia coli | 80 | 76 | Escherichia coli ESBL | 3 | 2 |
| Escherichia coli AmpC | 1 | 1 | Hafnia alvei | 1 | 0 |
| Escherichia coli BRMO | 3 | 3 | Hafnia alvei ESBL | 1 | 0 |
| Escherichia coli ESBL | 5 | 6 | Klebsiella oxytoca | 3 | 5 |
| Hafnia alvei | 2 | 0 | Klebsiella pneumoniae | 6 | 5 |
| Hafnia alvei ESBL | 1 | 0 | Morganella morganii | 3 | 3 |
| Klebsiella oxytoca | 4 | 6 | Morganella morganii BRMO | 1 | 0 |
| Klebsiella pneumoniae | 7 | 11 | Proteus mirabilis | 5 | 6 |
| Morganella morganii | 3 | 4 | Proteus mirabilis BRMO | 0 | 1 |
| Morganella morganii BRMO | 1 | 0 | Proteus vulgaris | 2 | 0 |
| Proteus mirabilis | 7 | 9 | Providencia stuartii | 0 | 1 |
| Proteus mirabilis BRMO | 1 | 1 | Providencia stuartii BRMO | 0 | 1 |
| Proteus vulgaris | 4 | 0 | Pseudomonas aeruginosa | 24 | 10 |
| Providencia stuartii | 0 | 1 | Pseudomonas aeruginosa BRMO | 1 | 0 |
| Providencia stuartii BRMO | 0 | 1 | Pseudomonas putida groep | 1 | 0 |
| Pseudomonas aeruginosa | 30 | 18 | Pseudomonas species | 1 | 0 |
| Pseudomonas aeruginosa BRMO | 3 | 0 | Pseudomonas species BRMO | 1 | 0 |
| Pseudomonas putida groep | 1 | 0 | Serratia marcescens | 9 | 4 |
| Pseudomonas species | 1 | 0 | Serratia marcescens BRMO | 1 | 0 |
| Pseudomonas species BRMO | 1 | 0 | Serratia marcescens ESBL | 2 | 0 |
| Raoultella species | 0 | 1 | Serratia species | 5 | 6 |
| Serratia marcescens | 11 | 6 | Sphingomonas paucimobilis | 6 | 0 |
| Serratia marcescens BRMO | 1 | 0 | Stenotrophomonas maltophilia | 5 | 7 |
| Serratia marcescens ESBL | 2 | 0 | **Total** | **176** | **118** |
| Serratia species | 6 | 7 |  |  |  |
| Sphingomonas paucimobilis | 7 | 0 |  |  |  |
| Stenotrophomonas maltophilia | 6 | 11 |  |  |  |
| **Total** | **241** | **195** |  |  |  |
|  |  |  |  |  |  |
| **All GNBs** ≥ 5 days | **Control** | **Intervention** | **All GNBs** ≥ 7 days | **Control** | **Intervention** |
| Achromobacter species | 0 | 2 | Achromobacter species | 0 | 1 |
| Acidovorax species | 2 | 0 | Acidovorax species | 2 | 0 |
| Acinetobacter baumannii | 0 | 1 | Acinetobacter baumannii | 0 | 1 |
| Acinetobacter pittii | 0 | 1 | Acinetobacter pittii | 0 | 1 |
| Alcaligenes faecalis | 1 | 0 | Alcaligenes faecalis | 1 | 0 |
| Burkholderia cepacia complex | 1 | 0 | Chryseobacterium indologenes | 3 | 0 |
| Chryseobacterium gleum | 0 | 1 | Citrobacter freundii | 1 | 1 |
| Chryseobacterium indologenes | 3 | 0 | Citrobacter freundii BRMO | 0 | 1 |
| Citrobacter freundii | 1 | 1 | Citrobacter species | 1 | 0 |
| Citrobacter species | 1 | 0 | Citrobacter species BRMO | 1 | 0 |
| Delftia acidovorans | 1 | 1 | Citrobacter species ESBL | 1 | 0 |
| Elizabethkingia species | 2 | 0 | Delftia acidovorans | 1 | 0 |
| Enterobacter aerogenes | 1 | 2 | Elizabethkingia species | 2 | 0 |
| Enterobacter cloacae complex | 8 | 5 | Enterobacter aerogenes | 1 | 1 |
| Escherichia coli | 19 | 14 | Enterobacter cloacae complex | 7 | 2 |
| Klebsiella oxytoca | 3 | 1 | Enterobacter cloacae complex ESBL | 5 | 0 |
| Klebsiella pneumoniae | 2 | 2 | Escherichia coli | 11 | 6 |
| Morganella morganii | 1 | 1 | Escherichia coli BRMO | 1 | 0 |
| Proteus mirabilis | 4 | 4 | Escherichia coli ESBL | 1 | 1 |
| Proteus vulgaris | 1 | 0 | Klebsiella oxytoca | 2 | 1 |
| Providencia stuartii | 0 | 1 | Klebsiella pneumoniae | 1 | 1 |
| Pseudomonas aeruginosa | 10 | 5 | Morganella morganii | 1 | 1 |
| Pseudomonas putida groep | 1 | 0 | Proteus mirabilis | 2 | 3 |
| Pseudomonas species | 1 | 0 | Providencia stuartii | 0 | 1 |
| Serratia marcescens | 8 | 3 | Providencia stuartii BRMO | 0 | 1 |
| Serratia species | 3 | 5 | Pseudomonas aeruginosa | 5 | 2 |
| Sphingomonas paucimobilis | 6 | 0 | Pseudomonas aeruginosa BRMO | 1 | 0 |
| Stenotrophomonas maltophilia | 3 | 7 | Pseudomonas putida groep | 1 | 0 |
| Citrobacter freundii BRMO | 0 | 1 | Pseudomonas species | 1 | 0 |
| Citrobacter species BRMO | 1 | 0 | Pseudomonas species BRMO | 1 | 0 |
| Enterobacter cloacae complex BRMO | 1 | 0 | Serratia marcescens | 5 | 3 |
| Escherichia coli BRMO | 2 | 1 | Serratia marcescens BRMO | 1 | 0 |
| Providencia stuartii BRMO | 0 | 1 | Serratia species | 3 | 5 |
| Pseudomonas aeruginosa BRMO | 1 | 0 | Sphingomonas paucimobilis | 4 | 0 |
| Pseudomonas species BRMO | 1 | 0 | Stenotrophomonas maltophilia | 1 | 5 |
| Serratia marcescens BRMO | 1 | 0 | **Total** | **68** | **38** |
| Citrobacter species ESBL | 1 | 0 |  |  |  |
| Enterobacter cloacae complex ESBL | 5 | 0 |  |  |  |
| Escherichia coli ESBL | 1 | 2 |  |  |  |
| Serratia marcescens ESBL | 2 | 0 |  |  |  |
| **Total** | **99** | **62** |  |  |  |
|  |  |  |  |  |  |
| **All GNBs** ≥ 10 days | **Control** | **Intervention** | **All GNBs** ≥ 14 days | **Control** | **Intervention** |
| Acidovorax species | 1 | 0 | Acidovorax species | 1 | 0 |
| Alcaligenes faecalis | 1 | 0 | Alcaligenes faecalis | 1 | 0 |
| Chryseobacterium indologenes | 3 | 0 | Chryseobacterium indologenes | 3 | 0 |
| Citrobacter freundii | 1 | 1 | Citrobacter freundii | 1 | 1 |
| Citrobacter species BRMO | 1 | 0 | Citrobacter species BRMO | 1 | 0 |
| Citrobacter species ESBL | 1 | 0 | Citrobacter species ESBL | 1 | 0 |
| Elizabethkingia species | 2 | 0 | Elizabethkingia species | 2 | 0 |
| Enterobacter aerogenes | 1 | 1 | Enterobacter aerogenes | 1 | 1 |
| Enterobacter cloacae complex | 5 | 1 | Enterobacter cloacae complex | 3 | 1 |
| Enterobacter cloacae complex ESBL | 3 | 0 | Enterobacter cloacae complex ESBL | 2 | 0 |
| Escherichia coli | 6 | 3 | Escherichia coli | 5 | 1 |
| Escherichia coli BRMO | 1 | 0 | Escherichia coli BRMO | 1 | 0 |
| Escherichia coli ESBL | 1 | 1 | Escherichia coli ESBL | 0 | 1 |
| Klebsiella oxytoca | 1 | 1 | Klebsiella oxytoca | 0 | 1 |
| Klebsiella pneumoniae | 1 | 1 | Klebsiella pneumoniae | 1 | 1 |
| Morganella morganii | 1 | 1 | Proteus mirabilis | 1 | 1 |
| Proteus mirabilis | 1 | 1 | Pseudomonas aeruginosa | 1 | 0 |
| Providencia stuartii | 0 | 1 | Pseudomonas aeruginosa BRMO | 1 | 0 |
| Pseudomonas aeruginosa | 2 | 1 | Pseudomonas putida groep | 1 | 0 |
| Pseudomonas aeruginosa BRMO | 1 | 0 | Pseudomonas species | 1 | 0 |
| Pseudomonas putida groep | 1 | 0 | Pseudomonas species BRMO | 1 | 0 |
| Pseudomonas species | 1 | 0 | Serratia species | 0 | 1 |
| Pseudomonas species BRMO | 1 | 0 | Stenotrophomonas maltophilia | 1 | 0 |
| Serratia marcescens | 1 | 0 | **Total** | **30** | **9** |
| Serratia species | 0 | 3 |  |  |  |
| Sphingomonas paucimobilis | 2 | 0 |  |  |  |
| Stenotrophomonas maltophilia | 1 | 1 |  |  |  |
| **Total** | **41** | **17** |  |  |  |

**Legend:** GNB identified in cultures that were taken of ICU patients with a length of stay of ≥2, ≥3, ≥5, ≥7, ≥10 and ≥14 days during the pre- and post-intervention period.
